# Prediction of Number of Cases of 2019 Novel Coronavirus (COVID-19) Using Social Media Search Index

**DOI:** 10.3390/ijerph17072365

**Published:** 2020-03-31

**Authors:** Lei Qin, Qiang Sun, Yidan Wang, Ke-Fei Wu, Mingchih Chen, Ben-Chang Shia, Szu-Yuan Wu

**Affiliations:** 1 School of Statistics, University of International Business and Economics, Beijing 100029, China; qinlei@uibe.edu.cn (L.Q.); sunqiang@uibe.edu.cn (Q.S.); wangyidanstats@163.com (Y.W.); 2 Graduate Institute of Business Administration, College of Management, Fu Jen Catholic University, New Taipei City 242, Taiwan; kefei000@yahoo.com.tw (K.-F.W.); 081438@mail.fju.edu.tw (M.C.); 3 Research Center of Big Data, College of management, Taipei Medical University, Taipei 110, Taiwan; stat1001@tmu.edu.tw; 4 College of Management, Taipei Medical University, Taipei 110, Taiwan; 5 Executive Master Program of Business Administration in Biotechnology, College of management, Taipei Medical University, Taipei 110, Taiwan; 6 Department of Food Nutrition and Health Biotechnology, College of Medical and Health Science, Asia University, Taichung 41354, Taiwan; 7 Division of Radiation Oncology, Lo-Hsu Medical Foundation, Lotung Poh-Ai Hospital, Yilan 265, Taiwan; 8 Big Data Center, Lo-Hsu Medical Foundation, Lotung Poh-Ai Hospital, Yilan 265, Taiwan; 9 Department of Healthcare Administration, College of Medical and Health Science, Asia University, Taichung 41354, Taiwan; 10 School of Dentistry, College of Oral Medicine, Taipei Medical University, Taipei 110, Taiwan

**Keywords:** social media, COVID-19, predictor, outbreak, new case

## Abstract

Predicting the number of new suspected or confirmed cases of novel coronavirus disease 2019 (COVID-19) is crucial in the prevention and control of the COVID-19 outbreak. Social media search indexes (SMSI) for dry cough, fever, chest distress, coronavirus, and pneumonia were collected from 31 December 2019 to 9 February 2020. The new suspected cases of COVID-19 data were collected from 20 January 2020 to 9 February 2020. We used the lagged series of SMSI to predict new suspected COVID-19 case numbers during this period. To avoid overfitting, five methods, namely subset selection, forward selection, lasso regression, ridge regression, and elastic net, were used to estimate coefficients. We selected the optimal method to predict new suspected COVID-19 case numbers from 20 January 2020 to 9 February 2020. We further validated the optimal method for new confirmed cases of COVID-19 from 31 December 2019 to 17 February 2020. The new suspected COVID-19 case numbers correlated significantly with the lagged series of SMSI. SMSI could be detected 6–9 days earlier than new suspected cases of COVID-19. The optimal method was the subset selection method, which had the lowest estimation error and a moderate number of predictors. The subset selection method also significantly correlated with the new confirmed COVID-19 cases after validation. SMSI findings on lag day 10 were significantly correlated with new confirmed COVID-19 cases. SMSI could be a significant predictor of the number of COVID-19 infections. SMSI could be an effective early predictor, which would enable governments’ health departments to locate potential and high-risk outbreak areas.

## 1. Introduction

A novel coronavirus, COVID-19 (formally known as 2019-nCoV), has emerged over the last few weeks since its outbreak in Wuhan City, China [1,2,3,4,5]. This severe acute respiratory syndrome (SARS)-like virus has infected over 75,000 people and killed over 2000 in China [1,2,3,4,5]. Case diagnoses have been confirmed in 26 countries, and 14 deaths have been reported outside of mainland China [1,2,3,4,5]. Currently, COVID-19 is spreading rapidly in South Korean communities, with almost 200 confirmed cases [1]. Little is known regarding this virus, aside from a possible incubation period of 2 to 14 days and a mortality rate of approximately 2.2% [5]. Increasing numbers of cases have also been reported in other countries across all continents except Antarctica, and the rate of new cases outside of China has outpaced the rate in China. These cases initially occurred mainly among travelers from China and those who have had contact with travelers from China [6,7]. However, ongoing local transmission has driven smaller outbreaks in some locations outside of China, including South Korea, Italy, Iran, and Japan, and infections elsewhere have been identified in travelers from those countries [8]. In the United States, clusters of COVID-19 with local transmission have been identified throughout most of the country [6,7].

COVID-19 is of critical concern for public health [9,10]. Health care providers should be updated regarding public health and COVID-19 outbreaks affecting their communities to promptly make correct decisions [10,11]. This would enable them to offer improved services in an efficient manner, which is crucial in the current situation [10]. Most health care providers depend on the Center of Disease Control and Prevention (CDC) to be informed on disease outbreaks or to be notified of new infectious COVID-19 [10]. However, we still do not have infectious diseases under control, especially novel COVID-19 [12]. Numerous researchers are attempting to gain an improved understanding of the evolution of COVID-19 and the causes of the disease [13,14,15]. This knowledge may help predict COVID-19 infections, which would allow a more targeted prediction of at-risk populations. Recently, social media search indices (SMSIs) have successfully indicated a correlation with the prediction of the transmission of infectious disease [16,17,18]. Studies have demonstrated that specific word searches in social networks may be a predictor of the transmission of influenza [18], SARS [17], dengue fever [19], and Middle East respiratory syndrome [16]. Nevertheless, SMSI was difficult to choose keywords, although they have a considerable effect on the performance of a prediction model. Since people continuously learn new terminology and change the search keywords they use, keywords should be updated regularly to maintain prediction performance [20]. As in the case of Google Flu, this system can fail to predict disease outbreaks correctly [21]. Therefore, the proposed digital surveillance system should be used with caution, or as a complementary method.

This study investigated the correlation between the number of new cases of COVID-19 and the search index for a popular social network in China, Baidu search index (BSI), as the reference SMSI. The aim of this study was to create an effective and affordable model to predict new cases, which would enable prompt and correct decision-making regarding public policies to limit the spread of COVID-19.

## 2. Individuals and Methods

### 2.1. Database

#### 2.1.1. Baidu Search Index in Social Media

Baidu is the most popular search engine in China and has accumulated a large amount of user behavior data since its establishment in 2000 [19,22]. The Baidu Index (http://index.baidu.com) is a data-sharing platform of Baidu’s behavioral data [19,22]. On this platform, users can obtain keyword search trends, gain insights into changes in personal needs, monitor media sentiment trends, locate digital consumer characteristics, and analyze market characteristics from an industry perspective. The BSI published on this platform reveals Internet users’ interest through changes in keyword searches. The index summates personal computer searches and mobile searches [19]. BSI was used as the representative SMSI and five keywords (in Chinese) related to suspected COVID-19 were selected, namely dry cough, fever, chest distress, coronavirus, and pneumonia, from 31 December 2019, to 9 February 2020. The optimal method of verifying the correlation between BSI and new confirmed COVID-19 was also selected and performed.

#### 2.1.2. Number of New Suspected Infection Cases

The National Health Commission (NHC) of the People’s Republic of China has been closely monitoring the epidemic situation since the Wuhan Health Commission announced an unexplained viral pneumonia notification. The pathogen of unexplained viral pneumonia was rapidly determined to be a new type of coronavirus [23,24]. The epidemic rapidly spread across the country and then across the world [25]. To ensure the distribution of accurate information, the NHC releases the latest data of COVID-19 cases, which include new and cumulative COVID-19 confirmed cases, suspected cases, serious cases, and death cases [24]. Dependent variables investigated in this study were the number of suspected COVID-19 cases and data from the latest briefing on COVID-19 cases in China released from 20 January 2020, to 9 February 2020, on the official website of the NHC. We also used our SMSI as a predictor to verify the correlation of COVID-19 confirmed cases from 31 December 2019, to 17 February 2020.

### 2.2. Method

#### 2.2.1. Model Formulation

The model considered in this study was as follows:

(1) $$log(Y_{t})=\mu +\overset{5}{\underset{p=1}{\sum }}\overset{10}{\underset{s=1}{\sum }}\beta_{s,p}log(X_{t-s,p})+\varepsilon_{t}$$

where  Y is the new COVID-19 case number, $X_{1},\cdots ,X_{5}$ are the BSI, μ is the constant, and ε is the error term. $X_{t-s,p}$ is the lagged time series (the lag order is *s*) of *p*th (*p* = 1–5) Baidu search indexes. Statistically, we should choose as many keywords as possible, but in our study, five keywords, dry cough, fever, chest distress, coronavirus, and pneumonia, are typical and adequate. The maximal lag order is 10 because the mean of incubation period is 10 days. Therefore, the superior limits of the two summations are 5 and 10, respectively. The COVID-19 case numbers were predicted by the lag series of BSI, and the coefficient β was estimated. 

#### 2.2.2. Parameter Estimation

The model contained 50 predictors. However, we only had 21 observations, which led to a typical high-dimensional problem in modern statistics. To select predictors, estimate parameters, and avoid overfitting, five methods were adopted to analyze the data, including subset selection, forward selection, ridge regression, lasso regression, and elastic net.

(1) Subset selection refers to the task of finding a small subset of available predictors that accurately predict the response. If the model has k predictors, then the subset selection method will choose the optimal model from the possible $2^{k}$ models, based on some criteria such as Akaike’s information criteria, Bayesian information criteria, or adjusted $R^{2}$.
(2) Forward selection is a stepwise selection method. It starts with no variables in the model, tests the addition of each variable using a chosen model fit criterion, adds the variable (if any), whose inclusion causes the most statistically significant improvement of the fit, and repeats this process until the model can no longer be improved to a statistically significant extent.
(3) Ridge regression is a method to create a parsimonious model when the number of predictor variables exceeds the number of observations, or when the data set has multi-collinearity. Employing the least-squares method is not possible when the number of predictors exceeds the number of observations, which leads to overfitting a model and the failure to find unique solutions. In contrast to the least-squares method, ridge regression shrinks parameters by L_2_ penalty, to obtain biased but lower variance estimators; thus, the estimates are reasonably reliable approximations of true population values. In this study, ridge regression solves the following problem:
  (2) $$Q=\sum_{t=1}^{T}{\left(log(Y_{t})-\mu -\sum_{p=1}^{5}\sum_{s=1}^{10}\beta_{s,p}log(X_{t-s,p})\right)}^{2}\;+\lambda \sum_{p=1}^{5}\sum_{s=1}^{10}\beta_{s,p}^{2}.$$
(4) Lasso regression is also a type of linear regression that uses shrinkage. Lasso regression performs both variable selection and parameter shrinkage by using the L_1_ penalty, which enhances the prediction accuracy and interpretability of the statistical model it produces. The only difference between lasso regression and ridge regression is the penalty function. In this study, lasso regression solves the following problem:
  (3) $$Q=\overset{T}{\underset{t=1}{\sum }}{\left(log(Y_{t})-\mu -\overset{5}{\underset{p=1}{\sum }}\overset{10}{\underset{s=1}{\sum }}\beta_{s,p}log(X_{t-s,p})\right)}^{2}+\lambda \overset{5}{\underset{p=1}{\sum }}\overset{10}{\underset{s=1}{\sum }}\left|\beta_{s,p}\right|$$
(5) Elastic net is a regularized regression method that linearly combines the L_1_ and L_2_ penalties of the lasso and ridge methods. The elastic net method often outperforms the lasso but has a similar sparsity of representation. In this study, elastic net solves the following problem:
  (4) $$Q=\overset{T}{\underset{t=1}{\sum }}{\left(log(Y_{t})-\mu -\overset{5}{\underset{p=1}{\sum }}\overset{10}{\underset{s=1}{\sum }}\beta_{s,p}log(X_{t-s,p})\right)}^{2}+\lambda \left(1-\alpha \right)\;\overset{5}{\underset{p=1}{\sum }}\overset{10}{\underset{s=1}{\sum }}\beta_{s,p}^{2}/2++\lambda \alpha \overset{5}{\underset{p=1}{\sum }}\overset{10}{\underset{s=1}{\sum }}\left|\beta_{s,p}\right|$$

#### 2.2.3. Accuracy Metrics

Six accuracy metrics were used to compare the performance of different methods: root mean squared error (RMSE), mean absolute error (MAE), mean absolute percentage error (MAPE), Pearson correlation, and the correlation of increment between $\hat{Y}$ and Y.

(5) $$RMSE\left(\hat{Y},Y\right)=\left(1/T\right){\left[\sum_{t=1}^{T}{\left({\hat{Y}}_{t}-Y_{t}\right)}^{2}\right]}^{1/2},$$

(6) $$MAE\left(\hat{Y},Y\right)=\left(1/T\right)\sum_{t=1}^{T}\left|{\hat{Y}}_{t}-Y_{t}\right|,$$

(7) $$MAPE\left(\hat{Y},Y\right)=\left(1/T\right)\sum_{t=1}^{T}\left|{\hat{Y}}_{t}-Y_{t}\right|/Y_{t},$$

(8) $$Corr.of.increment=Corr\left({\hat{Y}}_{t}-{\hat{Y}}_{t-1},Y_{t}-Y_{t-1}\right)$$

## 3. Results

**Figure 1:**
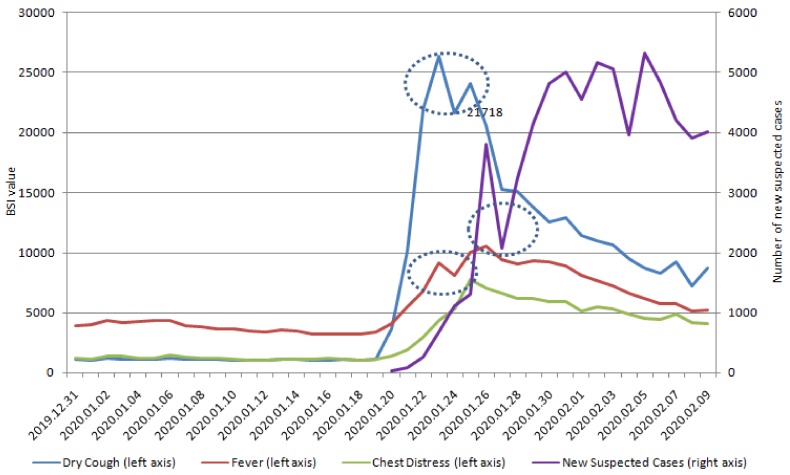
New suspected cases of COVID-19 and lag days of dry cough, fever, and chest distress.

**Figure 2:**
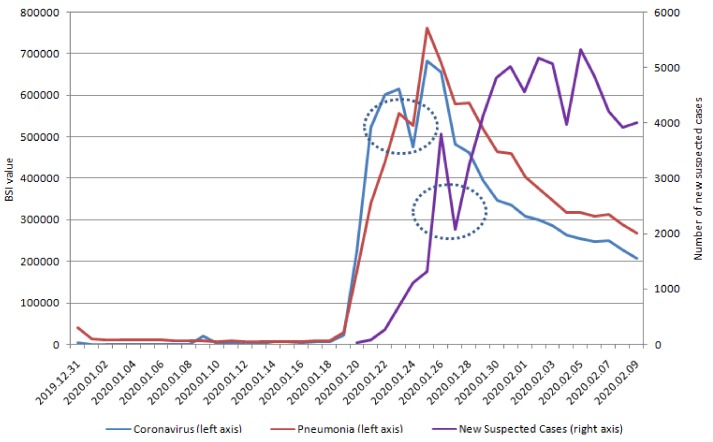
New suspected cases of COVID-19 and lag days of coronavirus and pneumonia.

We display the positive correlation between the series of new suspected COVID-19 cases and the lagged series of five keywords in BSI (Table 1). In addition, we identified a significant positive correlation between the lag days of BSI and new suspected COVID-19 cases, which revealed that changes in SMSI behaviors occurred earlier (6–9 days) than the confirmation of COVID-19 infection cases (Figure 1 and Figure 2). The correlation between new suspected COVID-19 case number and lag value in SMSI was statistically significant (Table 1). In our study, the SMSI was a predictor of new suspected COVID-19 infection confirmed cases and could be detected earlier by 6–9 days before the confirmation of new COVID-19 infection cases.

**Figure 3:**
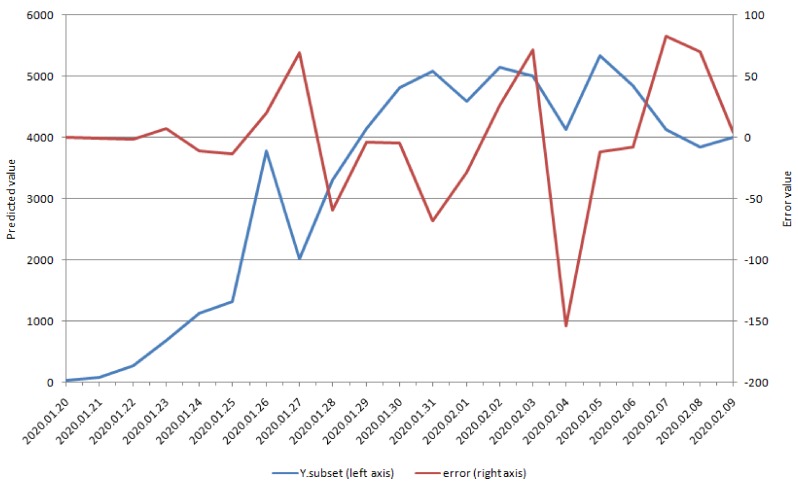
The prediction by subset selection and the error term.

Moreover, we summarize the accuracy metrics for five methods (Table 2). Among these methods, subset selection had the lowest RMSE, MAE, and MAPE and the highest correlation and correlation of increment, which indicated that it was the optimal method for explaining the data. The subset selection method only selected 10 of the 50 predictors. Figure 3 illustrates the prediction of the number of new COVID-19 cases and the error term. The prediction was close to the true series, and the error term was random and very small along the time axis, which confirmed that the subset selection method captured most of the relationship between search behaviors and the number of new COVID-19 cases.

Furthermore, we verified the optimal method of subset selection between the correlation and new confirmed COVID cases (Table 3). Table 1 reports significant correlations with SMSI on lag day 10 and new confirmed COVID-19 cases. The correlation between SMSI and new confirmed COVID-19 cases (nearly 50%) was lower than the correlation with new suspected COVID-19 cases (>80%; Table 1). The specific five keywords on lag 10 days were significantly correlated with new confirmed COVID-19 cases. The highest significant correlations, in order, were chest distress, fever, pneumonia, coronavirus, and dry cough on lag day 10. We also try to change some features (angina pectoris, difficulty urinating, impotence, urinary incontinence, dizziness) and compare the results with the original model results to illustrate the sensitivity of the model (Table A1). Because early symptoms do not include angina pectoris, difficulty urinating, impotence, urinary incontinence, or dizziness, we see no correlations between the lag time series of Baidu Indexes of these keywords. Based on these non-specific keywords, the overall estimation performance is worse with non-specific keywords. As a result, our prediction result is stable. Figure 1 and Figure 2 also demonstrate that the SMSI could be a predictor and detect COVID-19 cases, 10–12 days before they were confirmed.

We also identified similar patterns in SMSI and the series of new suspected and confirmed COVID-19 cases. Furthermore, the patterns appeared earlier in SMSI than in the series of new suspected and confirmed COVID-19 cases.

## 4. Discussion

Web and social media platforms have seen a rapid rise in user numbers, across both the developed and developing world [26]. Every day, millions of people self-report their symptoms online through social media, by using terms such as “fever,” “cough,” or “sore throat” [27]. Increasingly, people are using the Internet to search for information regarding their health [28]. An estimated 80% of all Internet users search for health information [29]. For instance, the number of tweets and searches related to an influenza-like illness increases during flu season. These anonymized data can help to track outbreaks across populations, almost instantaneously, and with geographically linked information [30]. Yahoo and Google have demonstrated that searches can detect outbreaks up to two weeks earlier than traditional disease surveillance [31]. The present study is the first to use BSI as the source of SMSI data in relation to COVID-19 epidemiology and investigate potential predictors of new suspected or confirmed COVID infection (Table 1 and Table 3). Tracking web data could allow a larger proportion of the population to be assessed, compared with traditional health surveillance methods [32].

**Figure 4:**
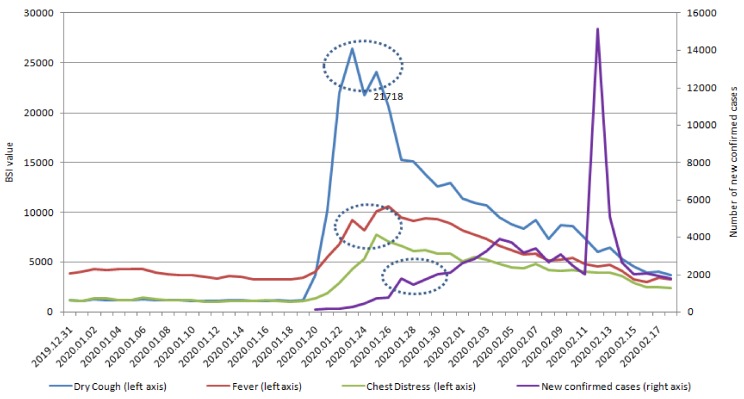
New confirmed COVID-19 cases and lag days of dry cough, fever, and chest distress.

**Figure 5:**
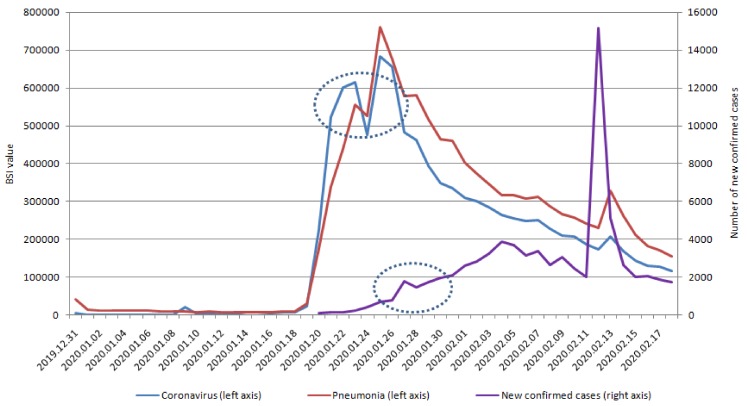
New confirmed COVID-19 and lag days of coronavirus and pneumonia.

Symptoms are not a diagnosis, and diseases can share common symptoms [33]. Therefore, accurate diagnosis or prediction of the underlying infectious agent remains the cornerstone of early warning systems, because it informs correct interventions [34]. SMSI-based models could serve as earlier, rapid, and affordable advanced sensing systems [35], which detect new suspected or confirmed COVID-19 infectious with specificity (Table 1, Table 3, Figure 1, Figure 2, Figure 4, Figure 5) and in real-time, enabling rapid and effective public health interventions.

Predicting new suspected or confirmed COVID-19 cases is crucial for developing targeted antiviral drugs, vaccines, or effective public health interventions, to prevent a future outbreak of COVID-19 [36]. In Table 1, the correlation between new suspected COVID-19 case numbers and lag value in SMSI was statistically significant. Changes in the SMSI could predict new suspected COVID-19 cases 6–9 days earlier. Moreover, our predictive method in SMSI was also significantly correlated with new confirmed COVID-19 10–12 days earlier (Table 3 and Figure 4 and Figure 5). The correlation was more than 80% between Lag value in SMSI and new suspected COVID-19 and nearly 50% with new confirmed COVID-19 cases. In Table 1, the correlations of coronavirus and pneumonia searches in social media were 0.8325 and 0.8130 (*p* value < 0.0001 and < 0.0001), respectively, nine days prior to the reporting of new suspected COVID-19 cases. Furthermore, dry cough, fever, coronavirus, and pneumonia searches were positively correlated with new suspected COVID-19 infections eight days earlier (Lag day 8; Table 1). The five keywords were all significantly correlated with new suspected COVID-19 cases, with correlation coefficients of 0.8288, 0.8896, 0.8396, 0.8301, and 0.8886 for dry cough, fever, chest distress, coronavirus, and pneumonia, respectively. The SMSI keyword search patterns occurred seven days before new suspected COVID-19 infection. The keyword search for fever and pneumonia was six days earlier than the new suspected COVID-19 cases, with over 90% correlation (Table 1). This SMSI could potentially be used to predict the areas and populations at risk of an outbreak of COVID-19. The SMSI in our study could be a predictor of COVID-19 infection, which would allow government health departments to formulate public health policies earlier and limit the spread of COVID-19 infection.

SMSI could be an effective and affordable tool for predicting emerging infectious diseases, and our findings in COVID-19 are compatible with studies on other emerging infectious diseases [35,37]. In Figure 1 and Figure 2, and Figure 4 and Figure 5, SMSI appeared to predict COVID-19 diagnosis a week early. Early prediction of COVID-19 infection benefits public health policies, by revealing specific infectious outbreak areas and at-risk populations, allowing governments to implement health policies to prevent the epidemic from expanding, as was the case with SARS [38]. Health authorities can educate highly susceptible populations in suspected infectious outbreak areas [38]. Public health policies may include the following: ensuring triage, early recognition, and source control (isolating patients with suspected COVID-19 infection); applying standard precautions for all patients; implementing empiric additional precautions (droplet, contact, and, airborne precautions, when necessary) for suspected cases of COVID-19 infection; implementing administrative controls; using environmental and engineering controls; and instructing the population not to eat raw eggs and to wash their hands with soap. The government should apply standard precautions for people who mention the five keywords (discomfort within 14 days) in SMSI. Standard precautions include hand and respiratory hygiene, the use of appropriate personal protective equipment, risk assessments, injection safety practices, safe waste management, proper linens, environmental cleaning, and sterilization of patient-care equipment [39]. Respiratory hygiene measures include ensuring that all patients cover their nose and mouth with a tissue or elbow when coughing or sneezing, offering medical masks to patients with suspected COVID-19 infection while they are in waiting in public areas or in cohort rooms, and exercising proper hand hygiene after contact with respiratory secretions [39]. If people have a history of long-term contact with birds, we suggest that they receive an influenza vaccine. We also recommend certain precautions for people who are highly susceptible to COVID-19 infection: consuming a balanced diet and exercising; not eating poultry eggs or products; never smuggling or purchasing meat from unknown birds; never touching or feeding migratory birds; never releasing or discarding birds; not mixing breeding birds with other poultry; and avoiding places with no air circulation or crowded places (such as traditional markets or hospitals, unless necessary). Moreover, SMSI may be more accurate in COVID-19 virus screening in highly suspected areas and populations; thus, government departments do not need to scramble for screening without specific targets, saving time, labor, and money for government health departments.

Table 2 summarizes different methods for the estimation of accuracy metrics in the highest correlation and incremental correlation. The last column of Table 2 presents the number of predictors after the application of the selection method. The number presented for the Ridge Regression is 50. We included the constant as a variable by mistake when calculating the number of variables, and corrected it in our manuscript. It does not mean that for each predictor the method relies on only two observations. Although the numbers of observation is less than the number of predictors, the application of these methods is correct, as they can handle the classical high-dimensional case. In our predictive model, subset selection was the optimal method for explaining the data. The subset selection method only selected 10 of the 50 possible predictors. Furthermore, the subset selection prediction of new suspected COVID-19 cases and the error term are displayed in Figure 3. The prediction in Figure 3 is close to the true series; the error term is random and very small along the time axis, which suggests that the subset selection method can capture most of the relationship between people’s search behavior and the new suspected COVID-19 case number. In our study, the highest correlation and incremental correlation in the subset selection model were 0.9996 and 0.9963, respectively. The intra-class correlation coefficient (ICC) is a robust correlation measure on section data, but our study is based on time series, so ICC may not be applicable. The highest correlation and incremental correlation were high enough to explain our model. Therefore, the subset selection method was optimal in our current predictor model, and our findings are compatible with those of previous studies [40,41].

Figure 1, Figure 2, Figure 4, Figure 5 display the outcomes of descriptive statistics. Figure 1 and Figure 2 illustrate that the keywords of fever and pneumonia were searched on social networks, six days before new suspected COVID-19 confirmed cases. The earliest keyword searches with a positive correlation over 80% were coronavirus and pneumonia, which was searched for nine days before new suspected COVID-19 cases. Using an SMSI to predict the outbreak of COVID-19 is affordable and effective and could be used to prevent people from hiding symptoms because they are afraid to seek medical attention, which may, in turn, lead to outbreaks.

This study is the first to investigate the possibility of using SMSI to predict outbreaks of COVID-19 in people in affected areas. The SMSI employed exhibited a high association with new suspected and confirmed COVID-19 cases. SMSI could be an effective early predictor, which would enable health government departments to locate potential and high-risk outbreak areas. Therefore, health government departments could prepare in advance for epidemic prevention and formulate new public health policies earlier.

This study has some limitations. First, people attempted to improve the accuracy of big data methods by, for instance, developing tools to overcome some of the problems that Google Flu Trends has recently encountered, including surges in media interest, which distorts the reported numbers of self-reported symptoms. COVID-19 is a novel infectious disease; thus, distorted reported numbers of self-reported symptoms may be unavoidable. Second, BSI is more popular than Google or Twitter use in China; thus, we have no other social network to validate our data. Therefore, the high usage rate of BSI in China is the principal corroborator of our conclusions. Third, statistically, early symptoms of COVID-19 are related to suspected patients, but not determining factors for new confirmed COVID-19 patients. New confirmed COVID-19 patients have been determined by the nucleic acid test. In addition, other respiratory diseases with similar symptoms might be the bias in the predictor model. Thus, the correlations between SMSI and new confirmed COVID-19 cases were lower than the correlation between SMSI and new suspected COVID-19 cases. Therefore, although the association between SMSI and new confirmed COVID-19 cases was strong, SMSI might be a good reference of potential outbreak of COVID-19, not a definitive tool for new confirmed COVID-19 cases.

## 5. Conclusions

Using SMSI to predict the outbreak of COVID-19 in populations in affected areas could be effective, and demonstrated a high correlation with new suspected and confirmed COVID-19 infection cases. SMSI could be an effective early predictor, which would enable health government departments to locate potential and high-risk outbreak areas. Therefore, health government departments could prepare in advance for epidemic prevention and formulate new public health policies earlier.

## Figures and Tables

**Table 1:** Correlation between new suspected Coronavirus disease 2019 (COVID-19) case number and lag value of five keywords in Baidu search index (BSI).

Variables	Dry Cough	Fever	Chest Distress	Coronavirus	Pneumonia
Lag 1 Day (*p* Value)	−0.1070	0.3586	0.6493	−0.2094	0.1922
	(0.6445)	(0.1105)	(0.0014)	(0.3623)	(0.4039)
Lag 2 Day (*p* Value)	0.1488	0.5650	0.7468	0.0626	0.4111
	(0.5198)	(0.0076)	(0.0001)	(0.7876)	(0.0641)
Lag 3 Day (*p* Value)	0.4183	0.7856	0.8590	0.3828	0.6517
	(0.0591)	(<0.0001)	(<0.0001)	(0.0868)	(0.0014)
Lag 4 Day (*p* Value)	0.5868	0.8596	0.9007	0.5847	0.7824
	(0.0052)	(<0.0001)	(<0.0001)	(0.0054)	(<0.0001)
Lag 5 Day (*p* Value)	0.6920	0.9147	0.9175	0.7352	0.8813
	(0.0005)	(<0.0001)	(<0.0001)	(0.0001)	(<0.0001)
Lag 6 Day (*p* Value)	0.7779	0.9124	0.8920	0.7831	0.9030
	(<0.0001)	(<0.0001)	(<0.0001)	(<0.0001)	(<0.0001)
Lag 7 Day (*p* Value)	0.8288	0.8896	0.8396	0.8301	0.8886
	(<0.0001)	(<0.0001)	(<0.0001)	(<0.0001)	(<0.0001)
Lag 8 Day (*p* Value)	0.8418	0.8361	0.7766	0.8795	0.8832
	(<0.0001)	(<0.0001)	(<0.0001)	(<0.0001)	(<0.0001
Lag 9 Day (*p* Value)	0.7758	0.7381	0.6935	0.8325	0.8130
	(<0.0001)	(0.0001)	(0.0005)	(<0.0001)	(<0.0001)
Lag 10 Day (*p* Value)	0.7077	0.6647	0.6044	0.7732	0.7306
	(0.0003)	(0.0010)	(0.0037)	(<0.0001)	(0.0002)

Table 1 reports the correlation between the current series of new confirmed case number and the lagged series of five Baidu indices (i.e., $Corr(N_{t},Index_{t-s})$, where $N_{t}$ is the new confirmed cases number, $Index_{t-s}$ where $N_{t}$ is the new confirmed case number, $Index_{t-s}$ is the lag, and s is the days/time series of the Baidu Index).

**Table 2:** Comparison of five methods for the estimation.

Variables	RMSE	MAE	MAPE	Correlation	Correlation of Increment	Number of Predictor
Subset Selection	51.6671	34.0739	0.0107	0.9996	0.9963	10
Forward Selection	70.0168	39.9790	0.0113	0.9993	0.9913	15
Ridge Regression	415.2922	279.6788	0.0827	0.9741	0.6937	51
Lasso Regression	519.7440	358.0979	0.1032	0.9597	0.4858	9
Elastic Net(alpha = 0.2)	527.4250	360.9563	0.1085	0.9585	0.4831	24
Elastic Net(alpha = 0.4)	516.1075	347.5939	0.1041	0.9602	0.5037	18
Elastic Net(alpha = 0.6)	514.7714	347.7290	0.1036	0.9604	0.4906	14
Elastic Net(alpha = 0.8)	510.1201	348.5859	0.1033	0.9611	0.5023	11

**Table 3:** Correlation between new confirmed cases number and lag time series of five Baidu Indexes.

Variables	Dry Cough	Fever	Chest Distress	Coronavirus	Pneumonia
Lag 1 Day (*p* Value)	−0.2444	−0.1588	0.0852	−0.3125	−0.2046
	(0.1930)	(0.4020)	(0.6544)	(0.0927)	(0.2781)
Lag 2 Day (*p* Value)	−0.1130	−0.0186	0.1971	−0.1861	−0.0720
	(0.5523)	(0.9221)	(0.2964)	(0.3248)	(0.7055)
Lag 3 Day (*p* Value)	−0.0235	0.0479	0.2392	−0.0968	0.0276
	(0.9017)	(0.8014)	(0.2030)	(0.6108)	(0.8849)
Lag 4 Day (*p* Value)	0.0257	0.1169	0.2954	0.0144	0.1360
	(0.8929)	(0.5386)	(0.1130)	(0.9397)	(0.4737)
Lag 5 Day (*p* Value)	0.1299	0.2169	0.3900	0.1134	0.2269
	(0.4938)	(0.2496)	(0.0331)	(0.5506)	(0.2279)
Lag 6 Day (*p* Value)	0.1659	0.2663	0.3895	0.1863	0.2861
	(0.3809)	(0.1549)	(0.0334)	(0.3243)	(0.1253)
Lag 7 Day (*p* Value)	0.2190	0.3271	0.4128	0.2442	0.3368
	(0.2449)	(0.0776)	(0.0234)	(0.1934)	(0.0688)
Lag 8 Day (*p* Value)	0.2729	0.3757	0.4440	0.2891	0.3621
	(0.1446)	(0.0407)	(0.0140)	(0.1213)	(0.0493)
Lag 9 Day (*p* Value)	0.3422	0.4381	0.4879	0.3461	0.4061
	(0.0641)	(0.0155)	(0.0062)	(0.0610)	(0.0260)
Lag 10 Day (*p* Value)	0.3823	0.4666	0.4998	0.3843	0.4363
	(0.0371)	(0.0093)	(0.0049)	(0.0360)	(0.0159)

Table 3 shows the correlation between the current series of new confirmed cases number and the lagged series of five Baidu Indexes (i.e., $Corr(N_{t},Index_{t-s})$, where $N_{t}$ is the new confirmed cases number, $Index_{t-s}$ is the lag, and s is the days/time series of the Baidu Index).

## Appendix A

**Table A1:** Correlation between new-confirmed cases number and lag time series of five non-specific COVID-19 features.

Variables	Angina Pectoris	Difficulty Urinating	Impotence	Urinary Incontinence	Dizziness
Lag 1 Day (*p* Value)	0.3243	0.7197	0.7327	0.2646	0.8089
	(0.1515)	(0.2382)	(0.1137)	(0.2464)	(0.4781)
Lag 2 Day (*p* Value)	0.1428	0.6323	0.6309	0.0359	0.8702
	(0.5368)	(0.7821)	(0.4522)	(0.8772)	(0.1603)
Lag 3 Day (*p* Value)	0.0086	0.5699	0.6210	−0.0479	0.9599
	(0.9705)	(0.6870)	(0.1927)	(0.8367)	(0.9775)
Lag 4 Day (*p* Value)	−0.2584	0.3913	0.5375	−0.3196	0.9445
	(0.2581)	(0.0794)	(0.1120)	(0.1578)	(0.8720)
Lag 5 Day (*p* Value)	−0.4884	0.2344	0.3950	−0.4854	0.9082
	(0.0747)	(0.3065)	(0.5764)	(0.4257)	(0.0861)
Lag 6 Day (*p* Value)	−0.5826	0.1215	0.3021	−0.6054	0.8637
	(0.1156)	(0.5998)	(0.1833)	(0.1136)	(0.1561)
Lag 7 Day (*p* Value)	−0.6768	−0.0797	0.2362	−0.7190	0.8054
	(0.7438)	(0.7313)	(0.3026)	(0.9922)	(0.4460)
Lag 8 Day (*p* Value)	−0.7272	−0.1196	0.1444	−0.7358	0.7309
	(0.0965)	(0.6055)	(0.5322)	(0.3351)	(0.1172)
Lag 9 Day (*p* Value)	−0.6612	−0.3142	−0.0412	−0.7723	0.6429
	(0.9211)	(0.1654)	(0.8594)	(0.9945)	(0.1779)
Lag 10 Day (*p* Value)	−0.6386	−0.2417	−0.0971	−0.6962	0.5584
	(0.6418)	(0.2912)	(0.6754)	(0.6625)	(0.2485)

Table A1 shows the correlation between the current series of new confirmed cases number and the lagged series of five Baidu Indexes (ie. $Corr(N_{t},Index_{t-s})$, where $N_{t}$ is the new confirmed cases number, $Index_{t-s}$ is the lag and s is the days/time series of the Baidu Index).
